# Placenta, the Key Witness of COVID-19 Infection in Premature Births

**DOI:** 10.3390/diagnostics12102323

**Published:** 2022-09-26

**Authors:** Tina-Ioana Bobei, Romina-Marina Sima, Gabriel-Petre Gorecki, Mircea-Octavian Poenaru, Octavian-Gabriel Olaru, Anca Bobirca, Catalin Cirstoveanu, Radu Chicea, Oana-Maria Topirceanu-Andreoiu, Liana Ples

**Affiliations:** 1 Department PhD, IOSUD, “Carol Davila” University of Medicine and Pharmacy, 020021 Bucharest, Romania; 2 Department of Obstetrics and Gynecology, Carol Davila University of Medicine and Pharmacy, 050474 Bucharest, Romania; 3 “Bucur” Maternity, Saint John Hospital, 012361 Bucharest, Romania; 4 Faculty of Medicine, Titu Maiorescu University, 040441 Bucharest, Romania; 5 Department of Internal Medicine and Rheumatology, Carol Davila University of Medicine and Pharmacy, 050474 Bucharest, Romania; 6 Department of Pediatrics, “Carol Davila” University of Medicine and Pharmacy, 020021 Bucharest, Romania; 7 Pediatrics Department, “Maria Sklodowska Curie” Emergency Children Clinical Hospital, 041451 Bucharest, Romania; 8 Faculty of Medicine, “Lucian Blaga” University of Sibiu, 550024 Sibiu, Romania; 9 OncoTeam Diagnostic, 010719 Bucharest, Romania

**Keywords:** SARS-CoV-2, COVID-19, placenta, preterm birth

## Abstract

Adverse perinatal outcomes, such as increased risks of pre-eclampsia, miscarriage, premature birth, and stillbirth have been reported in SARS-CoV-2 infection. For a better understanding of COVID-19 complications in pregnancy, histopathological changes in the placenta, which is the interface between mother and foetus, could be the place to look at. The aim of this study was to determine placental histopathological changes and their role in preterm birth in pregnant women with SARS-CoV-2 infection. We performed a prospective, observational study in a COVID-only hospital, which included 39 pregnant women with SARS-CoV-2 infection and preterm birth compared with a control group of 39 women COVID-19 negative with preterm birth and a placental pathology exam available. The microscopic examination of all placentas revealed placental infarction (64.1% vs. 30.8%), decidual arteriopathy (66.7% vs. 23.1%), intervillous thrombi (53.8% vs. 38.5%), perivillous fibrin deposits (59% vs. 46.2%), inflammatory infiltrate (69.2% vs. 46.2%), chorangiosis (17.9% vs. 10.3%), and accelerated maturation of the villi (23.1% vs. 28.2%).

## 1. Introduction

The World Health Organization (WHO) declared in March 2020 a global pandemic of coronavirus disease 2019 (COVID-19), caused by SARS-CoV-2.

Until August 2021, the total number of cases had surpassed 500 million and led to more than 6 million deaths. There is an ongoing effort to understand the transmission, disease pathogenesis and the short and long-term impact following infection.

It is estimated that pregnant women infected with SARS-CoV-2 have a 40% higher risk of experiencing pregnancy complications and adverse newborn outcomes [1].

Hormonal and immunological adaptations during pregnancy, as well as physiological and anatomical changes in the respiratory system, make pregnant women more vulnerable to several infections, including SARS-CoV-2 [2,3].

SARS-CoV-2 has surprised us, because although it is a severe respiratory virus, it seems to cause extrapulmonary manifestations, such as heart, liver, digestive tract, neurological disorders, and, last but not least, placental lesions [4].

The placenta acts like a physiological barrier between foetal and maternal blood flow, formed by the fusion of embryonic and maternal components attached to the uterine wall [5].

SARS-CoV-2 has been shown to bind via angiotensin II-converting enzyme (ACE2) to the cell membrane of target host cells, facilitated by the transmembrane serine protease type II transmembrane protease type S (TMPRSS2) initiation protease. ACE2 is expressed in most organs, including the placenta, in syncytiotrophoblast, cytotrophoblast, decidual stromal cells, endothelial smooth muscle cells, vascular cells, and decidual perivascular cells [6]. The coexpression of ACE2 and TMPRSS2 receptors in the placenta may increase the vulnerability of the placenta and foetus to SARS-CoV-2 infection [7].

The aim of this study was to identify the placental histopathological changes and their preterm birth impact in SARS-CoV-2-associated pregnancies.

## 2. Materials and Methods

A prospective cohort observational study (according to the STROBE Statement) was conducted, in which were included pregnant women with SARS-CoV-2 infection. Patients were recruited from the Bucur Maternity Hospital, a tertiary centre exclusively for COVID-19-positive patients. Recruitment was performed from March 2020 to June 2021. The patients were included after obtaining written informed consent and ethical committee approval for the study.

The inclusion criteria were: spontaneous pregnancy, gestational age at birth between 24 and 36 weeks, RT-PCR test positive for SARS-CoV-2, live foetus (echographic foetal activity), and placental pathology exam available.

The exclusion criteria were: in vitro fertilisation (IVF), foetal intrauterine, previous premature birth, cervical incompetence, presence of pessary or cerclage, congenital malformations and the denial of different investigations or treatment.

Maternal age, an obstetrical history of abortion, parity, the rupture of membranes, pregnancy-associated pathologies, birth gestational age, days of infection, birth delivery mode, COVID-19 symptoms, maternal and newborn evolution, CRP level, lymphocytes count, placental weight, and placental histopathological changes were the studied variables. All variables were obtained from the patient’s observation file.

After applying the inclusion and exclusion criteria, 39 pregnancies were selected.

The results of the patients with SARS-CoV-2 infection and premature birth were compared with one control group that included pregnant patients with premature birth without SARS-CoV-2 infection hospitalized between March 2018 and March 2020, so we could establish a connection between placental SARS-CoV-2-induced changes and the premature birth increased rate in SARS-CoV-2 infection. The placentas were examined routinely macroscopically and microscopically.

The statistical analysis was performed using Statistical Package for Social Sciences (SPSS) v. 19.0 (IBM Corp., Armonk, NY, USA). *p*-value < 0.05 (Pearson correlation) was considered statistically significant.

## 3. Results

The study included 39 parturients positive for SARS-CoV-2 infection with preterm delivery, and a control group formed by 39 non-COVID-19 parturients with placental pathology exam available.

The general and obstetrical characteristics of the studied groups were similar, but we can see there was a significant difference between the rates of premature rupture of membranes with a higher percentage in the non-COVID group (Table 1).

The percentage of caesarean delivery was higher in the COVID-19 period due to foetal distress (33.3%) and severe maternal clinical conditions (25.6%).

The maternal respiratory status was mild to severe: 53.8% were symptomatic, 23.1% required oxygen, 17.9% of patients were intubated, and there were no maternal deaths in our centre, although we had no information about the eight patients who were transferred for clinical degradation to ICU hospitals. Apgar scores of liveborn infants at 1 min were under 5 (10.3%) between 5 and 8 (61.6%), 9 and 10 (28.2%)—Table 2.

All newborns tested negative for SARS-CoV-2 at 24 h and 48 h by nasopharyngeal and throat swab.

A microscopic examination of all placentas revealed placental infarcts, decidual arteriopathy, intervillous thrombi, perivillous fibrin deposits, inflammatory infiltrate, chorangiosis, and accelerated maturation of the villi.

These findings were all present in varying degrees but were consistently seen in placentas. Comparatively, similar evidence of maternal malperfusion was also observed in the control group, but in much lower, statistically significant percentages (Table 3). Other microscopic findings in both populations included no histopathological abnormalities (5.1% in the COVID-19 group, 10.3% in the control group, respectively).

**Figure 1:**
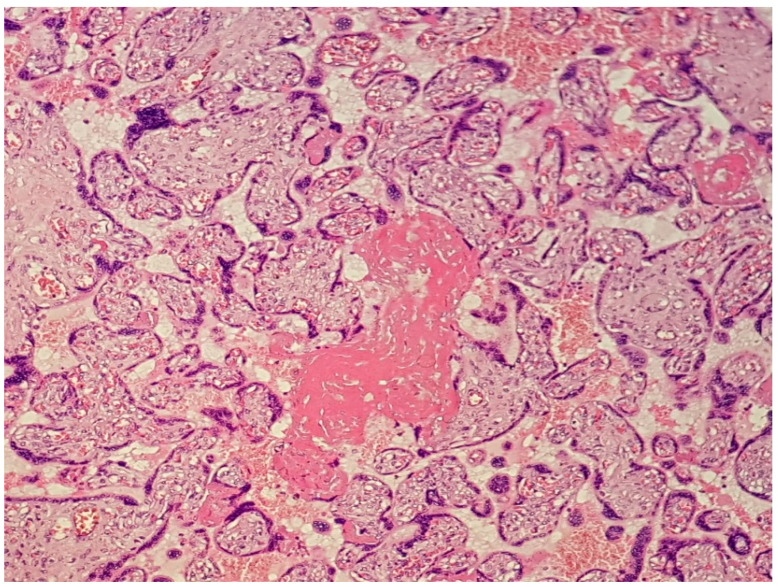
(Haematoxylin–eosin 10×). Presence of perivillous and intervillous fibrin with confluent aspects.

**Figure 2:**
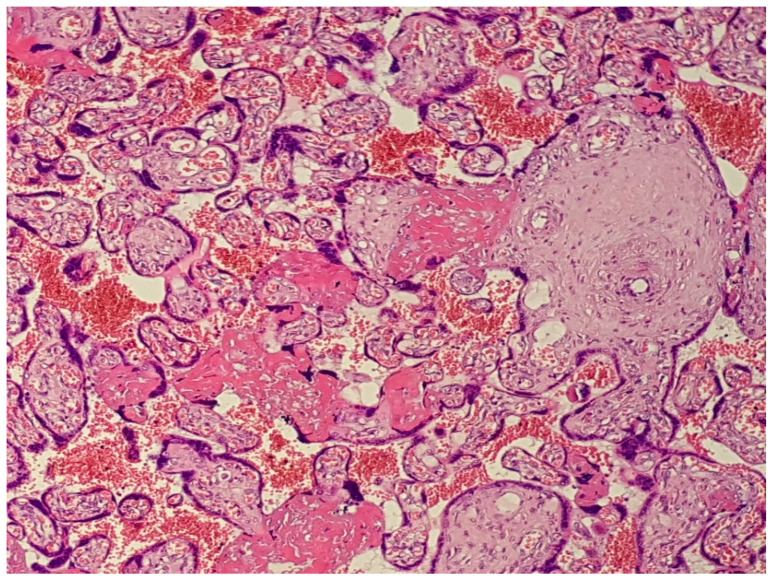
(Haematoxylin–eosin 10×). Features of accelerated maturation of the distal villi with numerous and prominent syncytial knots, fibrinoid deposits with incipient villous agglutination, and erosion of the syncytiotrophoblastic contour.

**Figure 3:**
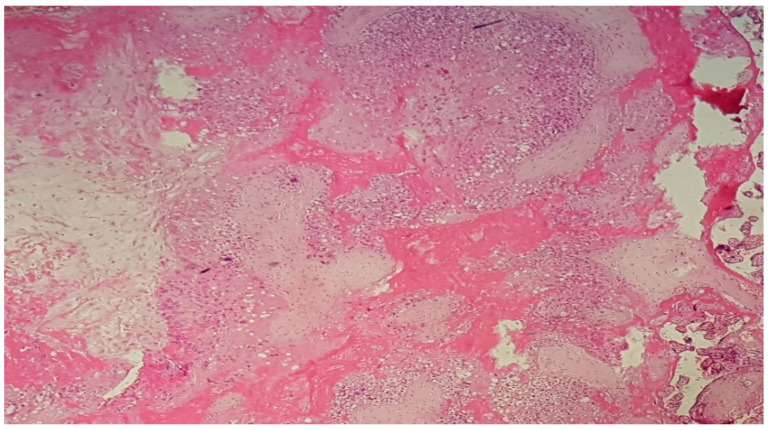
(Haematoxylin–eosin 10×). Extensive decidual ischemic changes and infarction with important fibrin deposition and inflammatory changes.

**Figure 4:**
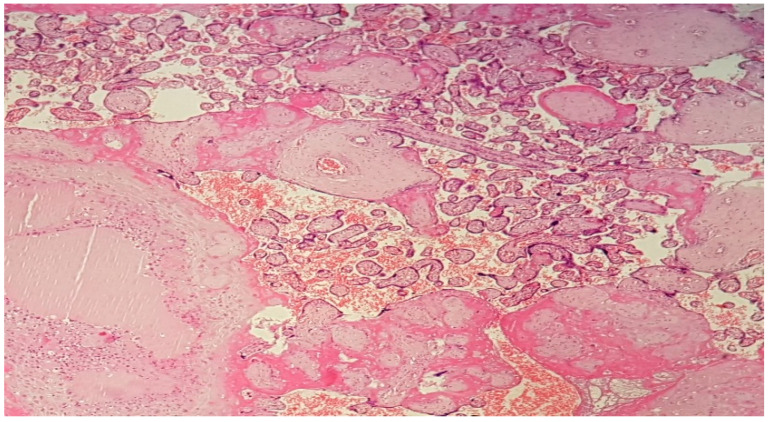
(Haematoxylin–eosin 10×). Decidual marked dystrophic and ischemic changes secondary to focal hypertrophic type arteriopathy; important perivillous noninflammatory fibrin deposits with encasement of necrotic and ghostlike chorionic villi; intervillous extravasated maternal blood.

**Figure 5:**
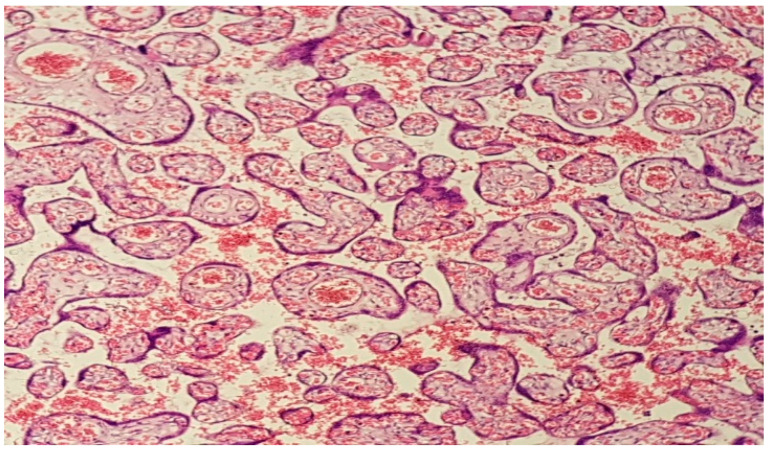
(Haematoxylin–eosin 10×). Chorangiosis shows excess numbers of villous capillaries lacking a surrounding, continuous layer of pericytes in the terminal villi.

However, the most common microscopic findings in placentas from patients with a confirmed SARS-CoV-2 infection were of the maternal vascular malperfusion (MVM) type, such as placental infarcts, decidual arteriopathy, perivillous fibrin deposits and chorangiosis (Figure 1, Figure 2, Figure 3, Figure 4 and Figure 5).

In the group of pregnant women with SARS-CoV-2 infection, a strong correlation was established between the presence of decidual arteriopathy and intubation (*p*-value = 0.040) and between the presence of intervillous thrombi and oxygen therapy (*p*-value = 0.016). In the study group, an inverse relationship was found between gestational age and the presence of MVM type changes (placental infarcts, *p*-value = 0.038; decidual arteriopathy, *p*-value = 0.16), between gestational age and chorangiosis (*p*-value = 0.010), but also between the Apgar score at 1 min and chorangiosis (*p*-value = 0.001) or intervillous thrombi (*p*-value = 0.010).

Unfortunately, we did not have the possibility in our centre to study the placentas by electronic microscopy or perform SARS-CoV-2 PCR tests.

## 4. Discussion

According to the latest studies, pregnancy has a significant influence in the outcome of SARS-CoV-2 infection, especially if associated with comorbidities such as pre-eclampsia or diabetes [8]. Pregnancy-specific physiological changes in the cardiorespiratory and immunological systems may be responsible for the increased morbidity among pregnant women, compared to the general population [9,10,11,12].

Still, the factors supporting the association between SARS-CoV-2 infection and adverse pregnancy outcomes remain unclear, despite growing research on clinical situations involving infected pregnant women [13].

Adverse perinatal outcomes have been reported, such as increased risks of pre-eclampsia, miscarriage, premature birth, and stillbirth [7].

In our study, the rate of caesarean section in the COVID-19 group of preterm labour increased (76.9%) in this period and two of the most frequent indications were the exacerbation of the symptoms, such as severe acute respiratory distress and foetal distress, in comparison with only 5.1% in the non-COVID-19 group with previous C-section and foetal distress as indications. As Bellos et al. found in their meta-analysis, SARS-CoV-2 pregnancies had a higher-than-expected rate of preterm and caesarean deliveries [14]. There are multiple studies that describe a direct association between COVID-19 and premature birth [15,16].

The purpose of this study was to identify placental histopathological SARS-CoV-2 changes and their role in preterm birth.

It is well known that placental tissue plays a critical role as an interface between mother and foetus, acting as a shield for the foetus against infection. Therefore, a deterioration in the mother’s clinical condition and in the placental structure may affect her ability to protect [17].

The most significant microscopic finding was an increase in the rate of MVM, especially placental infarcts, decidual arteriopathy, perivillous fibrin deposits, and chorangiosis, as Elishewa D. Shanes et al. found in their study of 16 placentas from patients with SARS-CoV-2 infection [18]. MVM has been related to poor perinatal outcome, such as oligohydramnios, IUGR (intrauterine growth restriction), premature birth, and stillbirth [19,20,21,22]. The major risk factors for MVM are gestational hypertension and preeclampsia [23,24], and in our study, only 30.8% of COVID-19 patients had associated pathologies, with 17.9% hypertensive disorders.

Obstetric complications, such as preterm birth, placental abruption, and foetal death in utero, can occur as a result of MVM lesions, with a higher prevalence rate in infected pregnant women than in a control group [25]. MVM lesions develop in pregnant women at variable degrees depending on the severity of infection, duration, and timing, which, in turn, may result in preterm birth [26]. There is a recognizable pattern of placental lesions associated with abnormal uterine perfusion. In addition, this can lead to many pathological variations, such as infarction of the villi, decidual thrombosis, intervillous thrombosis, vasculopathy, increased intervillous and perivillous fibrin, and accelerated maturation of the villi [27,28]. As our study revealed, the incidence of MVM lesions increased with the severity of SARS-COV-2 infection (oxygen therapy—*p* = 0.016, intubation—*p* = 0.040) and increased with decreasing gestational age (*p* < 0.05).

The viral agent responsible for SARS-CoV-2 infection enters the host cells by interacting with ACE2 (angiotensin-converting enzyme 2 receptor), expressed in the uterus and pregnant placenta. This hypothesis was reinforced by the increased prevalence of signs of decidual arteriopathy in pregnant women with SARS-CoV-2, suggesting a potential link between infection and the functional impairment of the placenta [29,30,31,32,33].

The probability of transplacental transmission was based on limited evidence, as the risk of vertical transmission is considered to be controversial [17]. The possible mechanisms of virus passage into the placenta are direct infection and rupture of syncytiotrophoblasts (STS), entry into trophoblasts or other placental cells through the endothelial microcirculation mediated by ACE2, ascending vaginal infection, and entry through the placental barrier of immune system cells of the infected pregnant woman [34]. In our study, no newborn tested positive for SARS-CoV-2 infection and no placentas or amniotic fluid were tested, as this rule was not included in the maternity protocol. Therefore, no mechanism of vertical transmission could be established, but considering the placental changes we can hypothesise that the placenta act as a shield against foetal infection but in the same time, those changes can impact the pregnancy and induce preterm birth, foetal distress, and poor neonatal outcome.

The study has some limits: a small number of included patients and the impossibility to perform electronic microscopy and RT PCR on the placentas. The pathologic examination of the placentas for all SARS-CoV-2 associated pregnancies and the availability of the premature births’ placentas for noninfected patients conferred strengths to the results. We propose to expand the study by comparing the placental changes also in term pregnancies with and without SARS-CoV-2 infection during pregnancy and to follow postnatal outcomes.

## 5. Conclusions

There have been several studies that have reported lesions specific to maternal vascular malperfusion occurring as histopathological changes induced by SARS-CoV-2 infection in the placenta.

Our study represents a report of MVM lesions from SARS-CoV-2 infection as a possible cause of preterm birth, especially in cases with severe infections. Since the SARS-CoV-2 virus continues to mutate and to infect millions of people including pregnant women, further studies are required to identity how it can impact the pregnancy and the newborn, and we believe that the placenta is the key witness in the process.

## Figures and Tables

**Table 1:** General and obstetrical characteristics in COVID-19 positive vs. non-COVID-19 patients with premature birth.

MATERNAL CHARACTERISTICS	COVID-19 PATIENTS (%)		NON-COVID-19 PATIENTS (%)		*p*-Value
**material age (years-percentage)**	18–30 y.o.	41	18–30 y.o.	53.8	0.164
	31–40 y.o.	46.2	31–40 y.o.	41	
	41–45 y.o.	12.8	41–45 y.o.	5.1	
**BMI (percentage)**	<18.5	7.7	<18.5	7.7	0.749
	18.5–24.9	69.2	18.5–24.9	69.2	
	25–30	12.8	25–30	17.9	
	>30	10.3	>30	5.1	
**gesta (number-percentage)**	1	33.3	1	43.6	0.581
	2	23.1	2	17.9	
	≥3	43.6	≥3	38.5	
**para (number-percentage)**	1	51.3	1	59	0.696
	2	28.2	2	28.2	
	≥3	20.5	≥3	12.8	
**monitored pregnancy (percentage)**	Yes	69.2	Yes	41	0.012
	No	30.8	No	59	
**gestational age of distribution (weeks-percentage)**	<28 w	7.7	<28 w	17.9	0.112
	28–31 w	12.8	28–31 w	17.9	
	32–36 w	79.5	32–36 w	64.1	
**premature rupture of membranes (percentage)**	YES	23.1	YES	51.3	0.010
	No	76.9	No	48.7	
**birth type (vaginal vs. caesarean-percentage)**	Vaginal birth	23.1	Vaginal birth	94.9	<0.001
	Caesarean birth	76.9	Caesarean birth	5.1	

**Table 2:** Characteristics of COVID-19-positive patients and newborns.

			*p*-Value
**symptomatic (percentage)**	Yes	53.8	0.000
	No	46.2	
**oxygen therapy (percentage)**	Yes	23.1	0.002
	No	76.9	
**intubation (percentage)**	Yes	17.9	0.006
	No	82.1	
**associated pathologies (percentage)**	Yes	30.8	0.001
	No	69.2	
**apgar scores (percentage)**	<5	10.3	0.000
	5–8	61.6	
	9	23.1	
	10	5.1	

**Table 3:** Comparison of microscopic features of COVID-19 patients vs. controls.

	COVID-19 PATIENTS	NON-COVID-19 PATIENTS	*p*-Value
**placental infarct (percentage)**	64.1	30.8	0.003
**perivillous fibrin deposits (percentage)**	59	46.2	0.263
**decidual artheriopathy (percentage)**	66.7	23.1	0.000
**intervillous thrombi (percentage)**	53.8	38.5	0.177
**chorangiosis (percentage)**	17.9	10.3	0.335
**accelerated villous maturation (percentage)**	23.1	28.2	0.610
**inflammatory infiltrate (percentage)**	69.2	46.2	0.040
**no histopathological abnormalities (percentage)**	5.1	10.3	0.384

## Data Availability

Writing to the correspondent author.

## References

[1] Metz T.D., Clifton R.G., Hughes B.L., Sandoval G.J., Grobman W.A., Saade G.R., Manuck T.A., Longo M., Sowles A., Clark K. (2022). Association of SARS-CoV-2 Infection With Serious Maternal Morbidity and Mortality From Obstetric Complications. JAMA.

[2] Bhatia P., Bhatia K. (2000). Pregnancy and the lungs. Postgrad. Med. J..

[3] Zhao X., Jiang Y., Zhao Y., Xi H., Liu C., Qu F., Feng X. (2020). Analysis of the susceptibility to COVID-19 in pregnancy and recommendations on potential drug screening. Eur. J. Clin. Microbiol..

[4] Zaim S., Chong J.H., Sankaranarayanan V., Harky A. (2020). COVID-19 and Multiorgan Response. Curr. Probl. Cardiol..

[5] Shang J., Wan Y., Luo C., Ye G., Geng Q., Auerbach A., Li F. (2020). Cell entry mechanisms of SARS-CoV-2. Proc. Natl. Acad. Sci. USA.

[6] Patel V.B., Zhong J.C., Grant M.B., Oudit G.Y. (2016). Role of the ACE2/angiotensin 1-7 axis of the renin-angiotensin system in heart failure. Circ. Res..

[7] Bloise E., Zhang J., Nakpu J., Hamada H., Dunk C.E., Li S., Imperio G.E., Nadeem L., Kibschull M., Lye P. (2020). Expression of Severe Acute Respiratory Syndrome Coronavirus 2 cell entry genes, angiotensin-converting enzyme 2 and transmembrane protease serine 2, in the placenta across gestation and at the maternal-fetal interface in pregnancies complicated by preterm birth or preeclampsia. Am. J. Obstet. Gynecol..

[8] Vouga M., Favre G., Martinez-Perez O., Pomar L., Acebal L.F., Abascal-Saiz A., Hernandez M.R.V., Hcini N., Lambert V., Carles G. (2021). Maternal outcomes and risk factors for COVID-19 severity among pregnant women. Sci. Rep..

[9] Di Mascio D., Khalil A., Saccone G., Rizzo G., Buca D., Liberati M., Vecchiet J., Nappi L., Scambia G., Berghella V. (2020). Outcome of coronavirus spectrum infections (SARS, MERS, COVID-19) during pregnancy: A systematic review and meta-analysis. Am. J. Obstet. Gynecol. MFM.

[10] Di Mascio D., Sen C., Saccone G., Galindo A., Grünebaum A., Yoshimatsu J., Stanojevic M., Kurjak A., Chervenak F., Suárez M.J.R. (2020). Risk factors associated with adverse fetal outcomes in pregnancies affected by Coronavirus disease 2019 (COVID-19): A secondary analysis of the WAPM study on COVID-19. J. Périnat. Med..

[11] D’Antonio F., Sen C., Di Mascio D., Galindo A., Villalain C., Herraiz I., Arisoy R., Ovayolu A., Eroğlu H., Canales M.G. (2021). Maternal and perinatal outcomes in high compared to low risk pregnancies complicated by severe acute respiratory syndrome coronavirus 2 infection (phase 2): The World Association of Perinatal Medicine working group on coronavirus disease 2019. Am. J. Obstet. Gynecol. MFM.

[12] Carbone L., Esposito R., Raffone A., Verrazzo P., Carbone I.F., Saccone G. (2020). Proposal for radiologic diagnosis and follow-up of COVID-19 in pregnant women. J. Matern. Neonatal Med..

[13] Baud D., Greub G., Favre G., Gengler C., Jaton K., Dubruc E., Pomar L. (2020). Second-Trimester Miscarriage in a Pregnant Woman With SARS-CoV-2 Infection. JAMA.

[14] Bellos I., Pandita A., Panza R. (2020). Maternal and perinatal outcomes in pregnant women infected by SARS-CoV-2: A meta-analysis. Eur. J. Obstet. Gynecol. Reprod. Biol..

[15] Wang X., Zhou Z., Zhang J., Zhu F., Tang Y., Shen X. (2020). A Case of 2019 Novel Coronavirus in a Pregnant Woman with Preterm Delivery. Clin. Infect. Dis..

[16] Mullins E., Evans D., Viner R.M., O’Brien P., Morris E. (2020). Coronavirus in pregnancy and delivery: Rapid review. Ultrasound Obstet. Gynecol..

[17] Sharps M.C., Hayes D.J., Lee S., Zou Z., Brady C.A., Almoghrabi Y., Kerby A., Tamber K.K., Jones C.J., Waldorf K.M.A. (2020). A structured review of placental morphology and histopathological lesions associated with SARS-CoV-2 infection. Placenta.

[18] Shanes E.D., Mithal L.B., Otero S., Azad H.A., Miller E.S., Goldstein J.A. (2020). Placental Pathology in COVID-19. Am. J. Clin. Pathol..

[19] Zhou Y.Y., Ravishankar S., Luo G., Redline R.W. (2020). Predictors of High Grade and Other Clinically Significant Placental Findings by Indication for Submission in Singleton Placentas from Term Births. Pediatr. Dev. Pathol..

[20] Khong T.Y., Mooney E.E., Ariel I., Balmus N.C., Boyd T.K., Brundler M.A., Derricott H., Evans M.J., Faye-Petersen O.M., Gillan J.E. (2016). Sampling and definitions of placental lesions: Amsterdam Placental Workshop Group Consensus Statement. Arch. Pathol. Lab. Med..

[21] Redline R.W. (2005). Severe fetal placental vascular lesions in term infants with neurologic impairment. Am. J. Obstet. Gynecol..

[22] Chen A., Roberts D.J. (2018). Placental pathologic lesions with a significant recurrence risk-what not to miss!. APMIS.

[23] Helfrich B.B., Chilukuri N., He H., Cerda S.R., Hong X., Wang G., Pearson C., Burd I., Wang X. (2017). Maternal vascular malperfusion of the placental bed associated with hypertensive disorders in the Boston Birth Cohort. Placenta.

[24] Weiner E., Feldstein O., Tamayev L., Grinstein E., Barber E., Bar J., Schreiber L., Kovo M. (2018). Placental histopathological lesions in correlation with neonatal outcome in preeclampsia with and without severe features. Pregnancy Hypertens..

[25] Aghaamoo S., Ghods K., Rahmanian M. (2021). Pregnant women with COVID-19: The placental involvement and consequences. J. Mol. Histol..

[26] Patberg E.T., Adams T., Rekawek P., Vahanian S.A., Akerman M., Hernandez A., Rapkiewicz A.V., Ragolia L., Sicuranza G., Chavez M.R. (2021). Coronavirus disease 2019 infection and placental histopathology in women delivering at term. Am. J. Obstet. Gynecol..

[27] Ernst L.M. (2018). Maternal vascular malperfusion of the placental bed. APMIS.

[28] Wong Y.P., Khong T.Y., Tan G.C. (2021). The effects of COVID-19 on placenta and pregnancy: What do we know so far?. Diagnostics.

[29] Algarroba G.N., Rekawek P., Vahanian S.A., Khullar P., Palaia T., Peltier M.R., Chavez M.R., Vintzileos A.M. (2020). Visualization of severe acute respiratory syndrome coronavirus 2 invading the human placenta using electron microscopy. Am. J. Obstet. Gynecol..

[30] Debelenko L., Katsyv I., Chong A.M., Peruyero L., Szabolcs M., Uhlemann A.-C. (2021). Trophoblast damage with acute and chronic intervillositis: Disruption of the placental barrier by severe acute respiratory syndrome coronavirus 2. Hum. Pathol..

[31] Hecht J.L., Quade B., Deshpande V., Mino-Kenudson M., Ting D.T., Desai N., Dygulska B., Heyman T., Salafia C., Shen D. (2020). SARS-CoV-2 can infect the placenta and is not associated with specific placental histopathology: A series of 19 placentas from COVID-19-positive mothers. Mod. Pathol..

[32] Levitan D., London V., McLaren R.A., Mann J.D., Cheng K., Silver M., Balhotra K.S., McCalla S., Loukeris K. (2021). Histologic and Immunohistochemical Evaluation of 65 Placentas From Women With Polymerase Chain Reaction-Proven Severe Acute Respiratory Syndrome Coronavirus 2 (SARS-CoV-2) Infection. Arch. Pathol. Lab. Med..

[33] Pique-Regi R., Romero R., Tarca A.L., Luca F., Xu Y., Alazizi A., Leng Y., Hsu C.D., Gomez-Lopez N. (2020). Does the human placenta express the canonical cell entry mediators for SARS-CoV-2?. Elife.

[34] Bukowska-Ośko I., Popiel M., Kowalczyk P. (2021). The immunological role of the placenta in SARS-CoV-2 infection-viral transmission, immune regulation, and lactoferrin activity. Int. J. Mol. Sci..

